# Supplementary material for: Neonatal hypothermia and adherence to World Health Organisation thermal care guidelines among newborns at Moi Teaching and Referral Hospital, Kenya
Source: PLoS One. 2021 Mar 23;16(3):e0248838. doi: 10.1371/journal.pone.0248838 (PMC7987163; doi:10.1371/journal.pone.0248838)
Supplement: Attachment — Submitted filename: Due to difficulty engaging another reviewer.docx [file pone.0248838.s002.docx]

Due to difficulty engaging another reviewer, I, as academic editor, added some comments to the first reviewer’s concerns regarding the report of postnatal hypothermia in a Kenyan hospital.

Dr. Lubuya et al go into details regarding the neonates encountering hypothermia. Thank you for collecting the data.

I agree with all of Reviewer 1’s comments with minor exceptions and a few additions.

Abstract: The abstract is too lengthy and basically gives a large part of the information of the paper. It needs to be shortened but can serve as a framework.

It would be helpful to know the normal birthweight range of infants born in Kenya, the gestational age and further common causes of neonatal death. Is hypothermia the lead cause? Is the hospital where the study took place well equipped? How long do mothers and babies stay there on average?

The authors are mentioning a birthweight of 200g on page 13, please correct.

Information given in the tables appears to be most useful and clear. Is it possible to transfer more information to a table?

Regarding language: There are grammatical errors i.e. Nigerian, Ethiopian instead of Nigeria and Ethiopia. The authors often use the word “while” instead of “and”. It would therefore help if a most proficient English speaker can review the paper with the authors. This team could also see where information can be shortened, transferred to a table and organized better.

In the right form the information reported will be useful for other countries and their efforts to improve the outcome of their neonates. It will in addition be helpful to agencies like the WHO and foundations focused on mother/baby survival.

While there is no need or opportunity to do a major revision, the revisions asked for by us though minor are numerous and demand a fair amount of work.
