# Supplementary material for: Neonatal hypothermia and adherence to World Health Organisation thermal care guidelines among newborns at Moi Teaching and Referral Hospital, Kenya
Source: PLoS One. 2021 Mar 23;16(3):e0248838. doi: 10.1371/journal.pone.0248838 (PMC7987163; doi:10.1371/journal.pone.0248838)
Supplement: Attachment — Submitted filename: Response to Reviewers _ Florence Lubuya 15122020.docx [file pone.0248838.s003.docx]

Journal Editorial Manager

PLOS One.

1160 Battery Street

Koshland Building East, Suite 225

San Francisco, CA 94111,

USA.

December 15, 2020

Dear Sir/Madam,

**REBUTTAL LETTER TO REVIEWER COMMENTS**

I would like to submit corrections for my manuscript entitled “**NEONATAL HYPOTHERMIA AND ADHERENCE TO WORLD HEALTH ORGANISATION THERMAL CARE GUIDELINES AMONG NEWBORNS AT MOI TEACHING AND REFERRAL HOSPITAL, KENYA**” by Prof. Winstone Mokaya Nyandiko, Dr. Paul Kiptoon and Dr. Florence Ajaya Lubuya.

***Comment 1: Did participants provide their written or verbal informed consent to participate in this study? b) If consent was verbal, please explain i) why written consent was not obtained, ii) how you documented participant consent, and iii) whether the ethics committees/IRB approved this consent procedure.***

***Response 1:*** The parents of the neonates provided written informed consent for all the participants and this informed consenting procedure was approved by the Institutional Research and Ethics Committee (IREC) of Moi University and Moi Teaching and Referral Hospital (Approval number: 0001484). The ethics statement has been updated including the approval number, clarification on written parental consent.

***Comment 2: In your Data Availability statement, you have not specified where the minimal data set underlying the results described in your manuscript can be found. PLOS defines a study's minimal data set as the underlying data used to reach the conclusions drawn in the manuscript and any additional data required to replicate the reported study findings in their entirety. All PLOS journals require that the minimal data set be made fully available. For more information about our data policy, please see*** [***http://journals.plos.org/plosone/s/data-availability***](http://journals.plos.org/plosone/s/data-availability)***.***

***Response 2:*** The dataset backing this study has been uploaded as supplementary information.

***Comment 3: Upon re-submitting your revised manuscript, please upload your study’s minimal underlying data set as either Supporting Information files or to a stable, public repository and include the relevant URLs, DOIs, or accession numbers within your revised cover letter. For a list of acceptable repositories, please see*** [***http://journals.plos.org/plosone/s/data-availability#loc-recommended-repositories***](http://journals.plos.org/plosone/s/data-availability#loc-recommended-repositories)***. Any potentially identifying patient information must be fully anonymized.***

***Response 3:*** The dataset backing this study has been uploaded as supplementary information (Florence Lubuya Dataset _PLoS ONE.xlsx).

***Comment 4: Introduction -This section provides a nice review of previous work done in this area and summarizes WHO guidelines. I think an additional paragraph describing the setting of this hospital, problems in this setting, and the attempts at instituting the guidelines would help the reader.***

***Response 4:*** This paragraph has been added in the methodology section describing the study setting (line 61-64) as “*At the time of data collection, the newborn unit had challenges offering appropriate thermal care equipment with only seven functional incubators in place. There was also a strain on space limiting the number of newborns considered for Kangaroo mother care.*”

***Comments 5: Materials and Methods - Well written and clear. Ethical approval was obtained from local committee. How was the adherence to warm chain steps collected? Was an unbiased observer present or was this based on nursing memory of what happened?***

***Response 5: “****Data on warm chain management was collected by independent observers who were trained research assistants. Other than observing, they also reviewed medical records and interviewed the newborn’s parent to collect sociodemographic data. All the collected data was cross-checked by the study’s investigators for consistency and accuracy****”*** (Line 64-68).

***Comment 6: Results - Demographics are listed, but it would be interesting to know what proportion of neonates with hypothermia had an underlying disease process like sepsis, etc.?
All other data and statistical tests appear to be accurate and appropriate for this type of report.***

***Response 6:*** Frequency and proportions of underlying conditions (diagnosis) are listed on table 1.

***Comment 7: Discussion - Clear and again a good review of previous studies with integration of the data from this report to support conclusions. Errors to correct. page 12, line 179: "There was sub-optimal adherence to the warm chain..." page 13, line 202: "2500g had a four-fold...."***

***Response 7:*** pages 12 and 13 have been modified to correct the phrasing on optimal adherence to warm chain guidelines and the risk of birth weight to day-1 neonatal mortality.

***Comment 8: Conclusion - Although conclusions are usually short, I do think this one could be expounded upon a little. I like the fact that the authors point to the fact that quality improvement strategies are needed to address the use of the warm chain. I think this is key and I hope a QI paper is forthcoming. Since the data from this study was collected in 2016, I hope this has taken place. Could you add a sentence or two addressing what might be implemented to prevent hypothermia?***

***Response 8:*** The conclusion section has been expanded as recommended by the reviewer. The improvements that have been witnessed over time at the newborn unit has been indicated in the conclusion and recommendations section. “*Although much improvement has been witnessed in the warm chain management strategies and infrastructure at MTRH, there is need to optimize the application of the warm chain guidelines quality improvement strategies addressed to the weak links identified in this study. An anticipatory approach to thermal care and priority triage of the preterm neonate admitted to newborn units should be adopted”* (Line 224-228).

***Comment 9: Overall, the information in this report further contributes to the knowledge that hypothermia leads to poor outcomes and that this early step is critical to the success of caring for newborns. It provides country specific information at a large, university hospital and identifies three factors that led to increased day one mortality. It also provides a good starting point for a QI project and report that can hopefully benefit other hospitals in a similar setting.***

***Response 9:*** Thank you for this comment.

***Comment 10: Abstract: The abstract is too lengthy and basically gives a large part of the information of the paper. It needs to be shortened but can serve as a framework.***

***Response 10:*** The abstract has been summarized as recommended.

***Comment 11: It would be helpful to know the normal birthweight range of infants born in Kenya, the gestational age and further common causes of neonatal death. Is hypothermia the lead cause? Is the hospital where the study took place well equipped? How long do mothers and babies stay there on average?***

***Response 11: “****Normal neonatal birthweight in Kenya ranges between 2500g to 3999g, with an appropriate gestational age of between 37 to 42 completed weeks****”*** (Line 43-45). Hypothermia is a major contributor to neonatal deaths occurring alongside other primary causes such as low gestational age (prematurity), birth asphyxia, congenital conditions warranting surgery and neonatal sepsis (Table 1). Most newborns spend less than 28 days in the newborn unit, however, the period may be extend based on the disease progression and treatment plan.

***Comment 12: The authors are mentioning a birthweight of 200g on page 13, please correct.***

***Response 12:*** This has been corrected to 2000g on line 199 (page 12).

***Comment 13: Information given in the tables appears to be most useful and clear. Is it possible to transfer more information to a table?***

***Response 13:*** All the tables have been updated and modified as per the PLOS ONE guidelines.

***Comment 14:*** ***Regarding language: There are grammatical errors i.e. Nigerian, Ethiopian instead of Nigeria and Ethiopia. The authors often use the word “while” instead of “and”. It would therefore help if a most proficient English speaker can review the paper with the authors. This team could also see where information can be shortened, transferred to a table and organized better.***

***Response 14:*** The entire document has been revised and grammatical errors corrected all through.

***Comment 15: In the right form the information reported will be useful for other countries and their efforts to improve the outcome of their neonates. It will in addition be helpful to agencies like the WHO and foundations focused on mother/baby survival.***

***Response 15:*** Thank you for this comment.

Sincerely,

**Florence Ajaya Lubuya,** MBchB, MMed Pediatrics (*Corresponding author*)

Department of Child Health and Pediatrics,

School of Medicine-Moi University

P.O. Box 4606-30100

**Tel:** +254702165151

**Email:** [flubuya@gmail.com](mailto:flubuya@gmail.com)
