# Supplementary material for: Neonatal hypothermia and adherence to World Health Organisation thermal care guidelines among newborns at Moi Teaching and Referral Hospital, Kenya
Source: PLoS One. 2021 Mar 23;16(3):e0248838. doi: 10.1371/journal.pone.0248838 (PMC7987163; doi:10.1371/journal.pone.0248838)
Supplement: Attachment — Submitted filename: Response Letter to the Reviewers_ Florence Lubuya 02032021.docx [file pone.0248838.s004.docx]

Journal Editorial Manager

PLOS One.

1160 Battery Street

Koshland Building East, Suite 225

San Francisco, CA 94111,

USA.

March 02, 2021

Dear Sir/Madam,

**REBUTTAL LETTER TO REVIEWER COMMENTS**

I would like to submit corrections for my manuscript entitled “**NEONATAL HYPOTHERMIA AND ADHERENCE TO WORLD HEALTH ORGANISATION THERMAL CARE GUIDELINES AMONG NEWBORNS AT MOI TEACHING AND REFERRAL HOSPITAL, KENYA**” by Prof. Winstone Mokaya Nyandiko, Dr. Paul Kiptoon and Dr. Florence Ajaya Lubuya.

**OVERALL COMMENT:**

***This is a very important report for neonatal care in Kenya and sub-Saharan Africa where such account is rare. Nyandiko, et al. report on adherence to WHO thermal care guidelines for newborns cared for a hospital in Kenya. They report on 372 newborns and find that 73.9% were hypothermic upon admission.***

We are grateful to the reviewers and editors for their comments on this manuscript.

**MINOR COMMENTS:**

***Comment 1: This is scientific report. Please add the aims in the introduction. The abstract has it. But it is lacking in the introduction.***

The study’s aims have been included in the last section of the background in the main manuscript.

***Comment 2: In the statistical section please correct the "Pearson chi-square" to "Pearson's chi-square". That is in on the abstract, the methods and the results.***

This correction has been made both in the abstract and methods section where Pearson's chi-square tests were mentioned.

***Comment 3: Table 1, Below the tables or somewhere put the meaning of the abbreviations. It needs to be a sort of clinician in English to understand and remember these abbreviations such MTRH, NBMS etc...***

The abbreviations on Table 1 have been explained in the legend section below the table. All other abbreviations used in the entire text have been fully defined the first time they are mentioned.

***Comment 4: Table 2, We do not need the "No (%)" column. I would recommend to remove this.***

The “No” column has been removed as advised by the reviewers.

***Comment 5: Table 5, There is seems to be very small cells there. The best chi-square here should be the Fisher's exact.***

Fischer’s exact test has been adopted for the results presented on table 5 (as advised by the reviewers).

***Comment 6: Table 6 is unnecessary. If the authors want keep it move to supplements.***

Table 6 has been removed as per the reviewer’s guidance.

***Comment 7: Figure 2 -Remove the no hypothermia [we can compute it by doing 100 - Hypothermia]***

The “*no hypothermia***”** graph has been removed from the figure 2.

***Comment 8: Figure 3 - I would not call it "cumulative". Strictly speaking "cumulative" plots are monotonic up i.e. they grow or stay constant. Use the x-axis title "number of warm chain steps adhered to" or something similar.***

The caption on Figure 3 has been changed to “*Number of warm chain steps adhered to*”

Sincerely,

**Florence Ajaya Lubuya,** MBchB, MMed Pediatrics (*Corresponding author*)

Department of Child Health and Pediatrics,

School of Medicine-Moi University

P.O. Box 4606-30100

**Tel:** +254702165151

**Email:** [flubuya@gmail.com](mailto:flubuya@gmail.com)
